# RNA-Binding Proteins and the Complex Pathophysiology of ALS

**DOI:** 10.3390/ijms22052598

**Published:** 2021-03-05

**Authors:** Wanil Kim, Do-Yeon Kim, Kyung-Ha Lee

**Affiliations:** 1 Division of Cosmetic Science and Technology, Daegu Haany University, Hanuidae-ro 1, Gyeongsan, Gyeongbuk 38610, Korea; wkim@dhu.ac.kr; 2 Department of Pharmacology, School of Dentistry, Kyungpook National University, Daegu 41940, Korea

**Keywords:** ALS, RNA-binding protein, membrane-less organelles

## Abstract

Genetic analyses of patients with amyotrophic lateral sclerosis (ALS) have identified disease-causing mutations and accelerated the unveiling of complex molecular pathogenic mechanisms, which may be important for understanding the disease and developing therapeutic strategies. Many disease-related genes encode RNA-binding proteins, and most of the disease-causing RNA or proteins encoded by these genes form aggregates and disrupt cellular function related to RNA metabolism. Disease-related RNA or proteins interact or sequester other RNA-binding proteins. Eventually, many disease-causing mutations lead to the dysregulation of nucleocytoplasmic shuttling, the dysfunction of stress granules, and the altered dynamic function of the nucleolus as well as other membrane-less organelles. As RNA-binding proteins are usually components of several RNA-binding protein complexes that have other roles, the dysregulation of RNA-binding proteins tends to cause diverse forms of cellular dysfunction. Therefore, understanding the role of RNA-binding proteins will help elucidate the complex pathophysiology of ALS. Here, we summarize the current knowledge regarding the function of disease-associated RNA-binding proteins and their role in the dysfunction of membrane-less organelles.

## 1. RNA-Binding Proteins and ALS

In 1939, the major league baseball player Lou Gehrig was diagnosed with amyotrophic lateral sclerosis (ALS), which is often called Lou Gehrig’s disease. The first descriptions of the disease in 1869 connected the symptoms and underlying neurological problems, and Charcot introduced the term ALS in 1874 [1]. ALS is a progressive neurodegenerative disease that affects nerve cells in the brain and spinal cord [2]. The progressive degeneration of motor neurons in ALS eventually leads to their death. Indeed, ALS is associated with complex pathophysiology including neuron loss, muscle wasting, excitotoxicity, microglial activation, peripheral inflammation, oxidative stress, mitochondrial dysfunction, endoplasmic reticulum stress, and synaptic remodeling [3,4,5,6,7,8,9,10,11]. There is no cure for ALS and disease progression is generally rapid. Eventually, after substantial respiratory and nutritional failure, death occurs in 70–80% of diagnosed individuals within 5 years [12]. The average length of survival from ALS onset to death is 2–4 years, and only 10% of ALS patients survive for more than 10 years [13]. To establish therapeutic strategies for treating ALS, the identification of disease-causing molecules and the unraveling of pathogenic pathways are required. Many putative exogenous factors have been investigated, including exposure to pesticides, viruses, cyanobacterial toxins, magnetic fields, heavy metals, a history of medical conditions, and lifestyle choices [12,14]. However, a definite environmental risk factor has not been clearly identified so far. Since the first identification of superoxide dismutase 1 (*SOD1*) as an ALS causative gene in 1993 [7], significant research efforts and advanced genetic approaches have identified mutations in more than 30 genes that cause ALS and frontotemporal dementia (FTD) [15,16,17,18]. In addition, 147 different gene mutations found in many different pathways have been shown to contribute to the pathogenesis of ALS [19,20]. Although sporadic ALS cases are much more common than familial ALS cases, a portion can be explained by ALS-causing mutations in genes. Indeed, some cases of ALS previously assumed to be sporadic have been subsequently identified as being caused by pathogenic mutations in ALS genes [17,21,22]. Interestingly, most ALS-causing mutation-harboring genes are closely related to RNA metabolism and form aggregates. Mutations in a series of RNA-binding protein genes, TAR DNA binding protein (*TARDBP*), FUS RNA-binding protein (*FUS*), TATA-box binding protein associated factor 15 (*TAF15*), EWS RNA-binding protein 1 (*EWSR1*), heterogeneous nuclear ribonucleoprotein A1 (*HNRNPA1*), *HNRNPA2B1*, Ataxin 2 (*ATXN2*), and TIA1 cytotoxic granule associated RNA-binding protein (*TIA1*), have been shown to cause or influence the disease risk for ALS and/or FTD [23,24,25,26,27,28,29,30,31]. ALS causative genes such as *FUS* and *TARDBP* encode RNA-binding proteins and lead to disrupted RNA metabolism [18]. ALS-causative mutations in *FUS* or *TARDBP* show abnormal stress granule formation with defects in translation, the formation of pathogenic RNA foci, the dysregulation of nucleocytoplasmic shuttling, as well as other forms of disrupted RNA metabolism [32,33]. The most common ALS- and FTD-causing gene mutation is a GGGGCC hexanucleotide repeat expansion in the first intron region of the chromosome 9 open reading frame 72 (*C9orf72*) gene [34,35]. ALS and FTD patients display several hundred to a few thousand copies of the GGGGCC repeat in the *C9orf72* gene [34], and the *C9orf72* repeat expansion’s toxicity increases with age, repeat length, and expression level [19,36]. Repeat-containing transcripts of the mutated *C9orf72* gene can form RNA foci enriched with RNA-binding proteins in induced pluripotent stem cell (iPSC)-derived neurons from ALS and FTD patients, as well as in motor neurons of C9orf72-ALS patients [37,38,39,40]. Even though conflicting evidence remains about the mechanisms of toxicity of *C9orf72* mutations [19,36,41], RNA foci are believed to sequestrate bound RNA-binding proteins and result in toxicity [42]. Dipeptide repeat (DPR), glycine-alanine (GA), glycine-arginine (GR), proline-arginine (PR), proline-alanine (PA), and glycine-proline (GP) are generated through repeat-associated non-ATG translation from hexanucleotide repeat expansion-containing transcripts [43,44,45,46]. Among the proposed disease-causing mechanisms of *C9orf72* mutations, the production and accumulation of DPR is considered to be one of the main contributors to its pathogenesis. Studies demonstrating the mechanisms of toxicity of DPRs in vitro and in Drosophila revealed that poly-GR and poly-PR DPRs interact with RNA-binding proteins or proteins that contain a low complexity sequence domain (LCD), also called the prion-like domain [47,48]. LCD is the key component that induces liquid-liquid phase separation and thereby mediates the formation of membrane-less organelles such as stress granules [23,47]. Indeed, poly-GR and poly-PR DPRs are most clearly associated with the nucleolus, a membrane-less organelle. Interestingly, the expression of poly-GR and poly-PR DPRs impairs the dynamics and function of interacting RNA-binding proteins that associate with numerous membrane-less organelles, including the nucleolus, stress granules, nuclear speckles, and Cajal bodies [47,49]. RNA-binding proteins have diverse functions; they can be involved in the post-transcriptional regulation of target genes and thus regulate their translation, mRNA degradation, and mRNA shuttling. Indeed, many RNA-binding proteins have liquid-liquid phase separation ability to form membrane-less organelles. The dysregulation of liquid-liquid phase separation is closely associated with several diseases such as dementia, ALS, and cancer [50,51,52,53].

**Figure 1:**
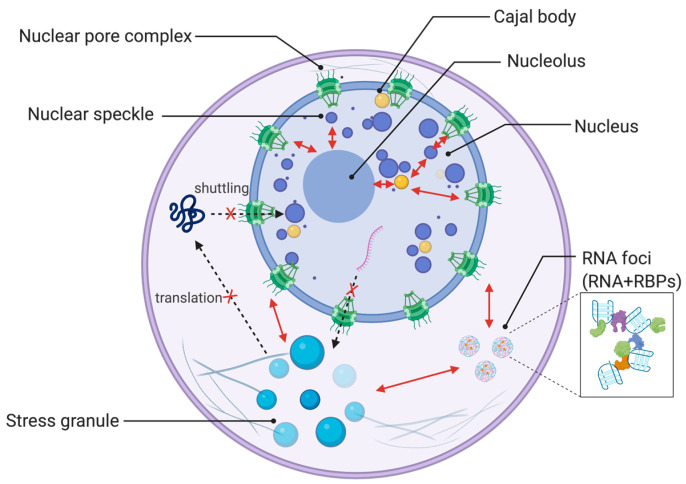
Membrane-less organelles that are affected by amyotrophic lateral sclerosis (ALS)-related RNA-binding proteins. Membrane-less organelles described in this review are illustrated, including the nucleolus, nuclear speckle, Cajal body, stress granule, along with RNA foci and nuclear pore complex. Dysregulation of RNA-binding proteins (RBPs) such as ALS-causing mutations leads to abnormal dynamics and function of the nucleolus, nuclear speckles, Cajal bodies, and stress granules. This dysfunction of membrane-less organelles is also interconnected to nucleocytoplasmic shuttling and RNA foci that may sequestrate RNA-binding proteins. These ALS-related cellular abnormalities influence each other. Created with BioRender.com.

Here, we focus on the cellular and functional features of ALS/FTD-related RNA-binding proteins, and discuss the complex pathophysiology of ALS by highlighting the role of RNA-binding proteins (Figure 1).

## 2. RNA-Binding Proteins and Membrane-Less Organelles

### 2.1. RNA-Binding Proteins and Disrupted Nucleocytoplasmic Shuttling

A defining characteristic feature of ALS and FTD is the loss of specific RNA-binding proteins from the nucleus and their mislocalization into cytoplasmic aggregates [54,55]. Emerging evidence suggests that impaired nucleocytoplasmic trafficking by the dysregulation of RNA-binding proteins is one of the key mechanisms causing ALS [56,57]. The cytoplasmic mislocalization and aggregation of the RNA-binding protein TARDBP is a common histopathological hallmark of ALS/FTD [58]. TARDBP pathology is present in about 97% of patients with ALS and up to 45% of patients with FTD [54,59]. Motor neurons in ALS show elevated poly(ADP-ribose) polymerase (PARP) activity and accelerated TARDBP aggregation in mammalian cells associated with neuronal death [60]. Aggregated and disease-causing mutations harboring TARDBP trigger the sequestration and disturbed localization of nucleocytoplasmic components such as nucleoporins and transport factors, as well as the disruption of nucleocytoplasmic shuttling [61]. Other nucleocytoplasmic transport factors are also recruited and sequestrated in the stress granules upon stress or treatment with ALS-implicated mutant proteins [62]. Although FUS pathology is uncommon and found in less than 1% of ALS and up to 9% of FTD cases [54,59], ALS-causing mutations in the nuclear localization signals of *FUS* also impair nucleocytoplasmic transport [57,63,64]. Although wild-type FUS normally localizes primarily in the nucleus, FUS has shown cytoplasmic mislocalization in ALS [65]. ALS-associated mutations in *FUS* impede its nuclear localization, and the increase in the altered localization pattern of mutant FUS in the cytoplasm correlates with rapid disease progression and a lower age of disease onset [16,66]. It has been proposed that the translocation of the transcript of splicing factor proline and glutamine-rich (SFPQ) accelerates the nuclear export of FUS, which can directly interact with *SFPQ* mRNA [65]. Mislocalized FUS mutants cause the mislocalization of snRNAs to the cytoplasm, which in turn causes a change in the behavior of the alternative splicing machinery [67]. ALS-associated FUS mutants that show the mislocalization and formation of cytoplasmic aggregates inhibit the splicing of minor introns by the sequestration of U11 and U12 small nuclear ribonucleoprotein (snRNP) [68]. Studies on TARDBP and FUS mislocalization show that either of these can affect the entire nucleo-cytoplasmic transport capability of the cells [19,69]. Several studies have indicated that the physical interaction between repeat RNA and RNA-binding proteins might also lead to the functional sequestration of RNA-binding proteins such as HNRNPA3, which plays a role in the cytoplasmic trafficking of RNA [70,71]. Several studies have reported a direct connection between *C9orf72* hexanucleotide repeat mutations and defects in nucleocytoplasmic shuttling [56,72]. These studies identified the co-aggregation of components of nucleocytoplasmic shuttling machinery with pathological protein aggregates or RNA foci. *C9orf72* hexanucleotide repeat expansion has been shown to cause nuclear import deficits in Drosophila and iPSC neurons, including an abnormal cytoplasmic accumulation of TARDBP [54]. Several RNA nuclear export factors, including ALYREF and GLE1, have been identified as genetic modifiers in the ALS-associated *C9orf72* model system [56]. The ALS-associated RNA-binding protein Matrin-3 (MATR3) was also found to colocalize with GGGGCC RNA foci in patient tissues as well as iPSC-derived motor neurons harboring the *C9orf72* mutation. Heaxanucleotide repeat expansion of *C9orf72* sequestered and perturbed the subcellular distribution of MATR3 in *C9orf72*-ALS patient-derived motor neurons [73].

*C9orf72*-derived DPR poly-GR and poly-PR have been shown to interact with several mediators of nucleocytoplasmic shuttling, including IPO5, IPO7, KPNA2, KPNB1, NUP205, XPO1, and TNPO1, as well as with RNA-binding proteins consisting of nuclear pore complexes, such as PARP1, YBX1, and LBR [47]. *C9orf72*-patient-iPSC-derived motor neurons have shown the mislocalization of U2 snRNP in the cytoplasm, and poly-GR as well as poly-PR are both associated with U2 snRNP [74]. The DPR-mediated dysfunction of U2 snRNP might explain mis-splicing events in patients with ALS/FTD [74]. Poly-GA also causes the mislocalization of HNRNPA3 by sequestration and leads to DNA damage [75]. The nuclear depletion of HNRNPA3 in fibroblasts derived from patients harboring *C9orf72* hexanucleotide repeats leads to an accumulation of pathological dipeptide repeat protein-containing inclusions [76]. ALS-causing mutations in the RNA-binding proteins *HNRNPA1* and *HNRNPA2B1*, especially in the prion-like domains of genes, promote cytoplasmic inclusion formation [26,77]. Several mutations in Profilin1 (*PFN1*), an actin-binding protein involved in actin cytoskeleton dynamics, have been identified in ALS [78,79], and mutant PFN1 has been shown to alter the distribution of RNA-binding proteins such as TARDBP, FUS, FMRP, and SMN, and disrupt the post-transcriptional regulation of their target mRNAs [80]. The modulation of actin homeostasis rescues nuclear pore instability and the dysfunction of RNA-binding proteins mediated by *C9orf72* repeat expansion or mutant PFN1 [80].

### 2.2. RNA-Binding Protein and Stress Granule Formation

Stress granules are a specific type of RNA granule and are composed of RNA and RNA-binding proteins, including repressed translational complexes. Stress granules are cytoplasmic members of the RNA granule family and are formed during cellular stress, particularly in neurodegenerative diseases and myopathies, resulting in dynamic membrane-less compartments [81]. Defects in both stress granule assembly and disassembly have been linked to neurodegenerative diseases [19,81,82]. Stress granules function as an RNA quality control system, stabilizing and editing mRNA, and arresting translation to prevent the accumulation of aberrant proteins [16,81]. Stress granules allow cells to cope with stress by stalling mRNA translation and moving synthesis towards cytoprotective proteins [23]. However, in disease-related stress conditions, stress granules can become persistent structures and act as a seed for the accumulation of RNA-binding proteins [81]. Stress granules have become one of the pathogenic phenotypes of ALS and FTD, and most ALS-causing mutations harboring RNA-binding proteins have a tendency to increase liquid-liquid phase separation and stress granule formation [23]. TARDBP in ALS has been reported to have a tendency to undergo liquid-liquid phase separation in vivo [33]; it forms amyloid-like aggregates in vitro and shows pathological oligomerization in vivo [33]. Indeed, in ALS and FTD, subsets of TARDBP-containing cytoplasmic inclusions were frequently positive for stress granule markers such as TIA-1, PABP, and eukaryotic initiation factor 3 (IF3) [83]. Although wild-type TARDBP localizes to stress granules under cellular stress conditions [84], the pathological RNA-binding protein TARDBP shows aggregation and accumulation in the spinal cords of patients with ALS [58] and leads to the formation of abnormal stress granules both in vitro and in vivo [16,85]. Conversely, mutations in *TIA-1* [28] or *ATXN2* [25], which are crucial components of stress granules [86,87], have also been identified as either causing ALS or increasing its risk, and ATXN2 altered the subcellular distribution and toxicity of TARDBP with enhanced mutant FUS toxicity [16,25,88]. Several ALS-related mutations in *TIA-1* have been identified [28,89,90], and disease-causing mutations delayed stress granule disassembly and promoted the accumulation of TDP-43 positive stress granules [28,91]. Mutations of *FUS* and *HNRNPA2B1* [26] have also led to the formation of aberrant stress granules. Wild-type FUS normally shows no re-localization from the nucleus to stress granules, but *FUS* mutation causes FUS to colocalize with stress granules that are larger and more numerous than wild-type FUS-mediated stress granules [92,93]. ALS-associated mutations in *HNRNPA1*, especially the Gly-rich low-complexity domain of HNRNPA1, have shown increased incorporation into stress granules [26,94]. Hexanucleotide expansions in the *C9orf72* gene impair the formation, dynamics, and function of stress granules [47,95], which might originate from the aberrant deposition of RNA-binding protein stress granule components, including phosphorylated TARDBP [96]. Poly-GR and poly-PR DPRs that arise from the *C9orf72* mutation can cause reduced stress granule internal dynamics, with HNRNPA1 and TIA1 phase separation [47] leading to translation repression [47,97]. Poly-GR and poly-PR DPR interact or colocalize with most of the stress granule component proteins, including G3BP1, TIA1, PABPC1, PTBP1, TARDBP, SFPQ, YBX1, HNRNPUL1, HNRNPA1, HNRNPA2B1, FXR1, LBR, SYNCRIP, HNRNPH1, and other RNA-binding proteins [47,98,99,100,101,102,103]. Mutations in *PFN1* cause not only defects in nucleocytoplasmic shuttling but also impairments in stress granule formation or clearance [80,104]. *SOD1* ALS mutations cause G3BP1-positive SOD1 aggregation in spinal cord motor neurons [92,105]. Mutant SOD1 is preferentially recruited to G3BP1 and TIA1-related protein (TIAR)-positive stress granules, and aberrant mutant SOD1-G3BP1 interaction delays stress granule assembly dynamics without direct binding to RNA according to the stress response [105,106,107]. Stress granule-recruited SOD1 induces changes in alternative splicing similar to mutations in *FUS* that cause ALS [108].

### 2.3. RNA-Binding Protein with Dysfunction of the Nucleolus and Cajal Bodies

The nucleolus is the largest nonmembrane-bound nuclear compartment and an important region for rRNA synthesis and the assembly of the eukaryotic ribosome [109]. Although the nucleolus is packed with a high density of protein and RNA, it is a dynamic structure with highly mobile constituents that can diffuse in and out of the nucleoplasm [110]. The role of liquid-liquid phase separation information of the nucleoli is increasingly recognized [47,110,111]. The nucleolus is one of the cellular stress sensors, and nucleolar stress, which disrupts nucleolar integrity by the dislocation of nucleolar proteins or abnormalities in the synthesis and processing of rRNA, is closely related to neurodegenerative diseases, including Alzheimer’s disease, Parkinson’s disease, Huntington’s disease, and ALS with DNA damage [109,110,112,113,114]. The disruption of the nucleolus or stress leads to cell cycle arrest, apoptosis, differentiation, or senescence [110]. Cellular analysis in different patients with ALS-FTD has shown altered nucleolar morphology and function, including swollen nucleoli and mislocalized nucleoproteins [109]. In the context of ALS-FTD, poly-GR and poly-PR DPRs generated from *C9orf72* mutations preferentially accumulate in the nucleoli and disrupt the transport of ribosomal proteins, rRNA processing, and ribosome assembly, leading to cell death [115,116,117]. These dipeptides, poly-GR, and poly-PR colocalize and interact with nucleolar proteins such as nucleolin (NCL), nucleophosmin 1 (NPM1), and fibrillarin (FBL) [47,109,118,119]. NPM1 translocates to the nucleoplasm depending on poly-GR and poly-PR and *C9orf72* transcripts [118,120], and NPM1 could act as an integrator of stress signals and determine cell fate according to diverse stresses [109]. NPM1 nucleoplasmic translocation and nucleolar stress induced by poly-GR or poly-PR expression have been shown to impair the dynamics of the nucleolus and decrease rRNA levels with translation efficiencies [47,109,118]. In particular, poly PR disperses and sequesters NPM1 from nucleoli and dissolved droplets. Poly-PR also entraps rRNA in static condensates [115]. Another nucleolar protein, NCL, is sequestered by GGGGCC repeated RNA originating from the *C9orf72* mutation and shows an altered distribution pattern, eventually inducing nucleolar stress [121,122,123]. Several studies have indicated that the physical interaction between repeat RNA and RNA-binding proteins might lead to the functional sequestration of RNA-binding proteins such as NCL, ADARB2, HNRNPA3, and HNRNPH [38]. In addition to the RNA-mediated sequestration of NCL, poly-GR and poly-PR from ALS-causing mutations of *C9orf72* also interact with NCL RNA-binding protein and disrupt its molecular dynamics in the nucleolus [47].

The nuclear bodies termed Cajal bodies are regions within the nucleus that are enriched in proteins and RNAs involved in mRNA processing. They are the main sites for the assembly of snRNPs. These Cajal bodies show frequent close proximity to the nucleolus but are structurally distinct from the nucleolus, although it can be difficult to clearly delimit the border between the two structures [124].

Toxic DPR poly-GR and poly-PR interact with Cajal body components such as RNA-binding proteins SRRM2 and XPO, which do not bind mRNA directly; however, other adaptor proteins act as a bridge to the interaction between XPO and mRNAs [47]. In live cells, the expression of poly-GR and poly-PR DPRs has been shown to reduce the mobile fractions of proteins that are associated with membrane-less organelles, including Cajal bodies [47,49]. DPRs, especially poly-GR, poly-PR, and poly-GA, have been associated with a strong change in the number of Cajal bodies [69]. ALS-linked FUS mutants show a decreased number of Cajal bodies [69], and *FUS* mutations induce the cytoplasmic accumulation of snRNAs and associated Sm proteins, leading to a decrease in the nuclear distribution of snRNPs in Cajal bodies [68,69]. The activation of the integrated stress response induces a profound reorganization of Cajal bodies, which is associated with the cytoplasmic assembly of stress granules and disturbance in the nuclear import of U-rich snRNPs [69]. Taken together, ALS-causing mutations and stress might disturb the nucleocytoplasmic shuttling of spliceosome components of snRNP and lead to abnormalities in RNA modification and ALS pathogenesis, with the disruption of telomerase biogenesis [125].

### 2.4. RNA-Binding Protein-Associated Dysregulation of Nuclear Speckle and Paraspeckle

Nuclear speckles, also known as interchromatin granule clusters, are membrane-less nuclear compartments enriched in pre-mRNA splicing and transcription factors located in the interchromatin regions of the nucleoplasm of cells [126]. Nuclear speckles are sites for splicing factor storage and modification with transcription, mRNA maturation, and export [127,128,129,130,131]. Alteration of the function of the composition of nuclear speckles leads to changes in alternative pre-mRNA splicing events as well as the disruption of diverse nuclear gene expression regulations [127]. Indeed, ALS and FTD are associated with a general disruption of nuclear structures and the disruption of nuclear speckles [127].

The self-organizing structure of nuclear speckles has dynamic liquid-like properties [47,127]. DPRs, especially poly-GR and poly-PR generated from *C9orf72* mutations, have also been shown to accumulate in nuclear speckles and disrupt their dynamics [47,97]. Poly-GR and poly-PR DPRs interact with splicing factor and the nuclear speckle component SRSF7, and significantly impact the dynamics of SRSF7 in nuclear speckles [47]. The alteration of liquid-like properties in nuclear speckles by DPRs induces specific splicing alterations in cells [132]. SRSF2, which interacts with TARDBP, FUS, and the hexanucleotide repeat expansion in *C9orf72*, has been shown to co-localize with *C9orf72* antisense RNA foci in *C9orf72* ALS patients [38,133]. SRSF2 also displays a nuclear punctate staining pattern with strong and larger speckles [133]. TARDBP protein has been shown to serve as a cellular scaffold for multiple nuclear bodies, including Cajal bodies and nuclear speckles [127,134]. In ALS, TARDBP forms intracellular inclusions that are mostly localized within the cytoplasm, leading to significant disruption of the nuclear structure and the function of nuclear compartments including nuclear speckles [127,134,135]. The dysregulation of *TARDBP* levels also impairs splicing events and especially disturbs the splicing of disease-associated transcripts [136]. The depletion of *TARDBP* decreases the expression of the nuclear speckle-specific noncoding RNA, *MALAT1* [137]. FUS is also found in nuclear speckles, and so mutations in *FUS* are expected to disrupt the structure and function of nuclear speckles [127].

Paraspeckles are mammalian-specific nuclear bodies that contain a long non-coding RNA, *NEAT1*, which acts as a seed for recruiting RNA-binding proteins and building a paraspeckle as well as more than 60 paraspeckle proteins [138,139]. Paraspeckles are involved in various aspects of the regulation of gene expression, and their role in many pathologies has been addressed [140]. A number of paraspeckle-enriched RNA-binding proteins, including SFPQ, FUS, EWSR1, TAF15, TARDBP, SS18L1, and HNRNPA1, are mutated in familial cases of ALS as well as other neurodegenerative diseases [17,24,141,142,143,144]. In spinal motor neurons during the early phase of ALS, TARDBP and FUS are enriched in paraspeckles and bound to *NEAT1* RNA [145]. Spinal neurons and glial cells in both sporadic and familial ALS with TARDBP pathology show enhanced paraspeckle formation. The loss and mutation of *TARDBP* accelerates paraspeckle assembly [146]. ALS-associated mutations in *TARDBP* diminished *NEAT1* non-coding RNA-mediated TARDBP liquid-liquid phase separation and resulted in a specific defect in the nuclear body and paraspeckle [147]. FUS is required for paraspeckle assembly [148], and ALS-associated mutations in *FUS*, especially in the low complexity domain, lead to the redistribution of FUS to the cytoplasm [149]. Mutation-harboring FUS also enhances dysfunctional paraspeckle formation through the accumulation of *NEAT1* non-coding RNA in cells [146,149]. GGGGCC repeat RNA-mediated RNA foci from *C9orf72* mutations are predominantly associated with paraspeckle proteins SFPQ, NONO, RBM14, FUS, and HNRNPH in cells and brain tissue in FTD [150]. ALS-associated poly-GR and poly-PR DPR interact with paraspeckle components such as HNRNPF, RNRPH1, HNRNPM, SFPQ, NONO, FAM98, RBM14, and MATR3 as well as *NEAT1* non-coding RNA [138,151]. Poly-PR DPR upregulates *NEAT1* expression and suppresses the function of HNRNPF and HNRNPH1, leading to increased paraspeckle formation and neuronal toxicity [138].

## 3. Conclusions and Future Directions

Although a limitation of this study includes the fact that we were not able to include all identified studies in the manuscript due to space restrictions, we have reviewed RNA-binding proteins in the context of ALS and highlighted how they are involved in many diverse membrane-less organelles. Most components of membrane-less organelles and ALS-related genes also have common features. As mentioned in this paper, many RNA-binding proteins have been directly or indirectly implicated in ALS and FTD.

Several membrane-less organelles have distinct function, composition, and localization but appear to be closely connected. It has been shown that the activation of the integrated stress response induces the accumulation of several factors into stress granules that are crucial mediators of nucleocytoplasmic transport, including import and export factors such as importin-β1, -β2, -α1, and Exportin-1, as well as nucleoporins, the core subunits of the nuclear pore complex [62,69]. Consequently, the induction of stress granule formation has a tendency to lead to the accumulation of nucleocytoplasmic shutting-related factors, which might lead to defects in nucleocytoplasmic trafficking.

With regard to the components of membrane-less organelles, one example, TARDBP, is one of the protein components of paraspeckles but is also found in stress granules, Cajal bodies, and in the nucleoplasm. Therefore, many RNA-binding proteins are components of several membrane-less organelles simultaneously. The interactome of ALS-associated genes shares somewhat similar RNA-binding proteins. Each interactome of the hnRNP family or key pathological genes is associated with ALS, including *TARDBP*, *C9orf72*, and *FUS* [151]. In the case of FUS, several hnRNPs, including HNRNPA1, R, and SYNCRIP, have been shown to co-accumulate in FUS-positive pathological inclusions [151,152]. Many other hnRNPs, including HNRNPD, L, and I, have also been found within FUS-negative pathological inclusions [151]. RNA foci derived from *C9orf72* hexanucleotide repeat expansion have been associated with HNRNPH1, HNRNPH3, HNRNPF, HNRNPA1, HNRNPA3, and other nuclear speckle related RNA-binding proteins [151,153]. The poly-GR and poly-PR interactome also somewhat similarly overlaps with other interactomes of ALS-related proteins. Additionally, RNA-binding proteins implicated in ALS might act co-operatively. Cytoplasmic inclusions of TARDBP increase HNRNPA1B protein levels, which harbors an elongated prion-like domain by the alteration of *HNRNPA1* pre-mRNA splicing and eventually leads to HNRNPA1B cytoplasmic accumulation [154]. We have summarized membrane-less organelles and the corresponding RNA-binding proteins that are components or have a functional relationship in Table 1.

Given this connectivity, a precise and systematic understanding of RNA binding might be important. Despite structural heterogeneity in RNA-binding proteins, many RNA-binding proteins have common functional roles. RNA-binding proteins can form dynamic and cooperative complexes with other RNA-binding proteins to fulfill their role in membrane-less organelles and in diverse RNA regulatory steps. Therefore, it is not at all surprising that so many RNA-binding proteins have been linked directly or indirectly to the pathogenesis of ALS. The molecular pathogenesis of ALS appears to be driven by various and complex inter-connected RNA metabolic processes that cannot be solely attributed to any one dysfunctional event. The dysregulation of one RNA-binding protein might disrupt the structure or function of a specific complex of RNA-binding proteins. This might disrupt the dynamics and function of membrane-less organelles that harbor a dysfunctional complex of RNA-binding proteins. An abnormality in one RNA-binding protein will also lead to an unusual RNA-binding protein complex that harbors an abnormal RNA-binding protein; this will eventually lead to the dysfunction of other membrane-less organelles as well as the malfunction of RNA metabolism, known as the domino or snowball effect. Given the diverse functional and structural role of RNA-binding proteins, the dysregulation of specific RNA-binding proteins might lead to diverse functional abnormalities and complex pathophysiology. The complex and various pathophysiologies of ALS might be linked directly or indirectly to each other because of RNA-binding proteins in some but not all cases.

For a better understanding of the complex and linked pathophysiology of ALS, the functional role of RNA-binding proteins could be important. In terms of the functional role of RNA-binding proteins, they generally bind to miscellaneous RNA transcripts and exhibit a widespread distribution in cells. The relationship between target mRNA and RNA-binding protein should also be considered in order to understand the exact function and importance of RNA-binding proteins in ALS. Knockdown or overexpression of specific RNA-binding proteins often shows exactly the opposite effect according to target mRNAs [91,151]. For example, SYNCRIP, which functions as a transacting factor for posttranscriptional regulation, has been shown to rhythmically accelerate mRNA degradation and increase non-canonical translation of several target genes [156,157]. PTBP1 is also known to be one of the components of the pre-mRNA splicing machinery, which is a multi-component complex necessary for the splicing step [158]. In addition to splicing regulation, PTBP1 also activates mRNA degradation but increases the translation of specific target genes [159,160]. Therefore, the dysfunction of specific RNA-binding proteins can lead to reduced mRNA degradation of target mRNA, but also more rapid mRNA degradation of the other target mRNA. Translation initiation, mRNA degradation, the nucleocytoplasmic shuttling of RNA, splicing, and many other RNA metabolism statuses could differ according to the target mRNA. Therefore, it is usually difficult to define the role of specific RNA-binding proteins as being singular. To understand these complex functional roles of RNA-binding proteins, one needs to consider the context of RNA-binding proteins such as target mRNA and status-dependent RNA-binding protein complex. In addition to target RNA of RNA-binding proteins, the opposite and various functions of RNA-binding proteins are based on the context of interacting proteins. These RNA-binding proteins usually form complexes with other RNA-binding proteins and RNAs for specific roles, and various functions of RNA-binding proteins are based on the context of the interacting proteins. RNA-dependent regulation can be modulated by multiple RNA and RNA-binding protein complexes that exchange their binding partners in response to the cellular environment. RNA-binding proteins generally bind to target RNAs and recruit their interacting proteins in different ways based on the cellular context. Context-dependent interactions of RNA-binding proteins with target transcripts lead to diverse consequences, resulting in functional divergence. To elucidate the roles of the given RNA-binding protein, context-dependent approaches should be considered.

Sporadic ALS cases are still more common than familial ALS cases, although some sporadic ALS cases have been identified as being caused by pathogenic mutations. It is thought that a complex and various interaction between genetic and environmental factors might be involved in sporadic ALS. Although RNA-binding protein abnormalities cannot account for all sporadic ALS cases, RNA binding proteins such as TARDBP, FUS, TAF15, EWSR1, HNRNPA1, HNRNPA2B1, MATR3, and TIA1 are strongly linked with sporadic ALS [17,24,77,89,90,91,161,162]. Furthermore, the global dysregulation of non-coding RNAs has been reported in motor neurons from people with sporadic ALS, and the generation and regulation of non-coding RNA are mediated by RNA binding proteins including ALS-associated RNA binding proteins [163,164]. There is also a possible association between some ALS-causing environmental factors and the resulting abnormal function in RNA binding proteins. Therefore, further studies on not only the ALS-causative mutation but also the functional abnormality of the RNA binding proteins according to the environmental determinants of ALS are necessary.

In the field of ALS, the reason why there is a selective affection of particular types of neurons in ALS is one of the more perplexing conundrums. Accumulating evidence demonstrates that neuronal stimulation and signal transmission require the trafficking and local translation of mRNA for proper neural function [165,166]. The upper and lower motor neurons are the longest cells in the body, and the transport of RNA and the localized translation of target mRNA are crucial for their proper function. RNA binding proteins, including ALS-associated RNA binding proteins, are involved in RNA transport and local translation. The relationship between the unique physiology of motor neurons and the function of RNA binding proteins might be one of the reasons for the susceptibility of motor neurons in ALS. Moreover, the brain shows unusually high levels of alternative splicing, including testis and liver [167]. Since RNA binding proteins discussed in this review are directly or indirectly involved in RNA splicing, the dysregulation of RNA binding proteins could mediate the improper function of the central nervous system by disrupting the diversity of gene expression in the neuron.

This information indicates that the context-dependent complex of RNA-binding proteins may warrant further study; it may be the key to understanding the complex, linked, and entangled pathophysiology of ALS. The elucidated map of RNA-binding proteins in ALS will shed light on strategies for the development of therapeutics for ALS.

## Figures and Tables

**Table 1:** Membrane-less organelles and the corresponding RNA-binding proteins.

Location	RNA-Binding Proteins	Function
Stress granules	FUS	Abnormal stress granule formation [32,93]
	TARDBP	Abnormal stress granule formation [33,85]; GR or PR DPR interactor [47,96]
	ATXN2	Stress granule components; cause ALS or increase its risk [16,25]
	TIA-1	Stress granule components; abnormal stress granule dynamics [28,91]; GR or PR DPR interactor [47]
	HNRNPA2B1	Formation of aberrant stress granules [26]; GR or PR DPR interactor [47,101]
	HNRNPA1	Increased incorporation into stress granules [94]; GR or PR DPR interactor [47,102]
	G3BP1	GR or PR DPR interactor [47,99]
	PABPC1	GR or PR DPR interactor [47,101]
	PTBP1	GR or PR DPR interactor [47,101]
	SFPQ	GR or PR DPR interactor [47]
	YBX1	GR or PR DPR interactor [47,101]
	HNRNPUL1	GR or PR DPR interactor [47]
	FXR	GR or PR DPR interactor [47]
	LBR	GR or PR DPR interactor [47]
	SYNCRIP	GR or PR DPR interactor [47,101]
	HNRNPH	GR or PR DPR interactor [47,101]
	PFN1	Impairments in stress granule dynamics [80,104]
	SOD1	Incorporation into stress granule; delaying stress granule assembly [105,106]
Nucleolus	NPM1	Colocalization and interaction with GR or PR DPR [47,109]; mislocalization with impaired nucleolus dynamics [118,120]
	NCL	Colocalization and interaction with GR or PR DPR also with repeat RNA [38,47,118,119], altered distribution, and nucleolar stress [122,123]
	FBL	Colocalization DPR [109,155]
	ADARB2	Interaction with repeat RNA of *C9orf72* [38]
	HNRNPA3	Interaction with repeat RNA of *C9orf72* [38]
	HNRNPH	Interaction with repeat RNA of *C9orf72* [38]
Cajal bodies	SRRM2	GR and PR DPR interactor [47]; DPR altered dynamics [69]
	FUS	Decreasing number of Cajal bodies [69]; altering distribution of snRNP into Cajal bodies [68,69]
Nuclear speckles	SRSF7	Altered dynamics by interaction with GR or PR DPR [47]
	SRSF2	Colocalize with *C9orf72* RNA foci [38,133]
	TARDBP	Disruption of nuclear speckles [127,135]; disturbing splicing event [136] and expression of MALAT1 [137]
	FUS	Disruption of nuclear speckles [127]
Paraspeckles	SFPQ	Paraspeckle-enriched and mutation in ALS [141,144], associated with *C9orf72* [138,150,151]
	FUS	Paraspeckle-enriched and mutation in ALS [141,143,145], dysfunction of paraspeckle [149], associated with *C9orf72* RNA foci [150]
	EWSR1	Paraspeckle-enriched and mutation in ALS [24,143]
	TAF15	Paraspeckle-enriched and mutation in ALS [141,143]
	TARDBP	Paraspeckle-enriched and mutation in ALS [141,143,145]; defect in paraspeckle [146,147]
	SS18L1	Paraspeckle-enriched and mutation in ALS [141,143]
	HNRNPA1	Paraspeckle-enriched and mutation in ALS [141,143]
	NONO	Associated with *C9orf72* RNA foci [150] and DPR [138,151]
	RBM14	Associated with *C9orf72* RNA foci [150] and DPR [138,151]
	HNRNPH	Associated with *C9orf72* RNA foci [150] and regulated by poly PR [138]
	HNRNPF	GR or PR DPR interactor [138,151] and regulated by poly PR [138]
	HNRNPM	GR or PR DPR interactor [138,151]
	FAM98	GR or PR DPR interactor [138,151]
	MATR3	GR or PR DPR interactor [138,151]
Nucleocytoplasmic shuttling	FUS	Dysregulation of nucleocytoplasmic shuttling [32,63]
	TARDBP	Dysregulation of nucleocytoplasmic shuttling [33,61]
	HNRNPA3	Nucleocytoplasmic transport capability [71]; mislocalization by DPR [75]
	MATR3	Sequestrated by RNA foci [73]
	PARP1	GR or PR DPR interactor; nucleocytoplasmic shuttling modulator [47]
	YBX1	GR or PR DPR interactor; nucleocytoplasmic shuttling modulator [47]
	LBR	GR or PR DPR interactor; nucleocytoplasmic shuttling modulator [47]
	U2 snRNP	Mislocalization by DPR [74]
	HNRNPA1	Cytoplasmic inclusion formation [26]
	HNRNPA2B1	Cytoplasmic inclusion formation [26]
	PFN1	Altered distribution of RNA-binding proteins [80]

## Data Availability

Not applicable.
